# Supplementary material for: Exploring GPCR‐arrestin interfaces with genetically encoded crosslinkers
Source: EMBO Rep. 2020 Sep 14;21(11):e50437. doi: 10.15252/embr.202050437 (PMC7645262; doi:10.15252/embr.202050437)
Supplement: Supplementary file 1 — Appendix [file EMBR-21-e50437-s001.pdf]

Appendix Böttke *et al.*

Table of Content:

Appendix Figure S1: Recruitment of Bpa-βarrestin mutants to the PTH1R as detected using the NanoBiT® reporter assay (SmBit-Arr/PTH1r-LgBit). Page 2

Appendix Figure S2: Immunoprecipitation (IP) of βarr1-D78Bpa-2xStrep with the Strep-Tactin® XT system and following LC/MS/MS analysis. Page 6

Appendix Figure S3: Plasmids used for the incorporation of ncAAs Page 7

A

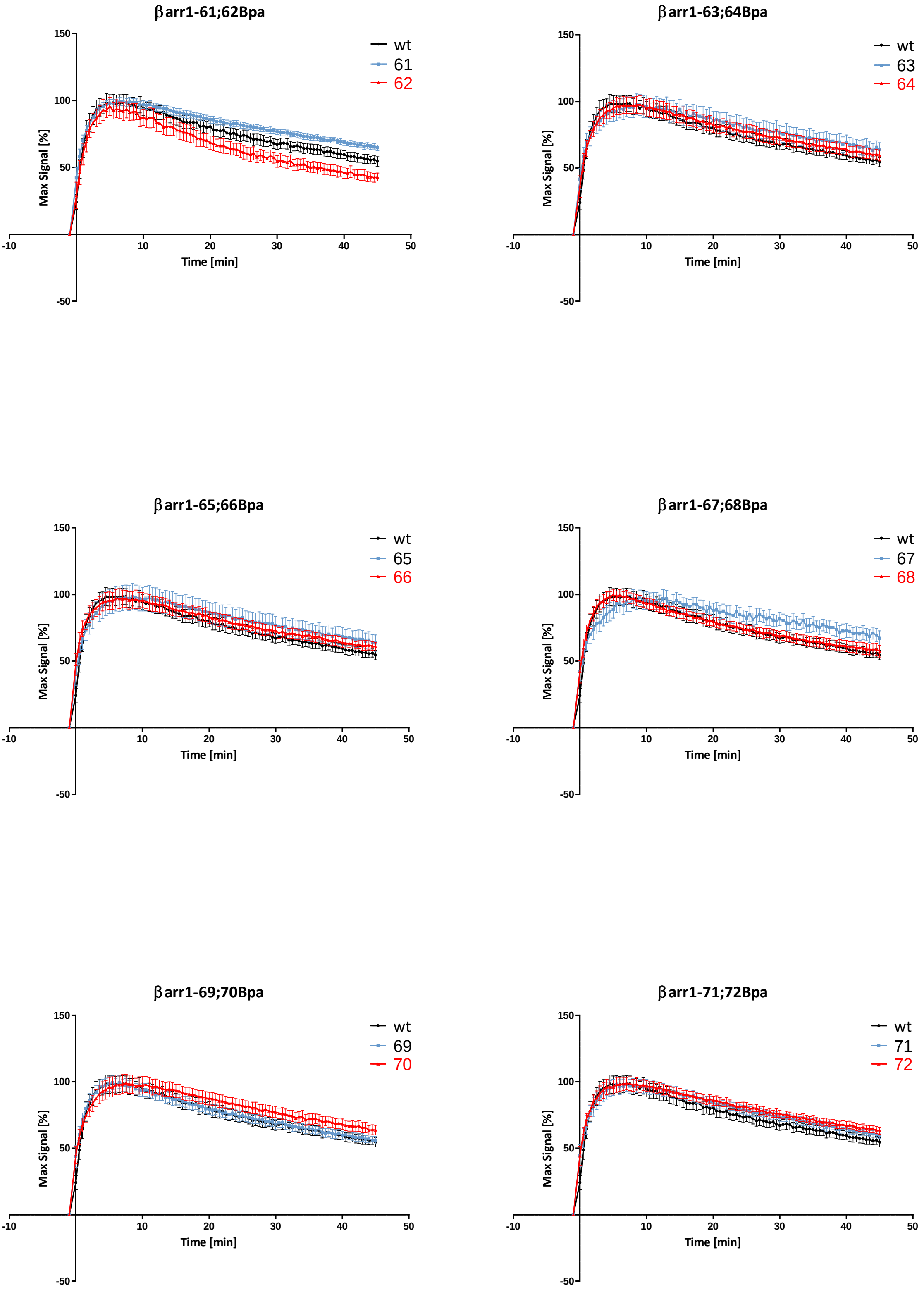
βarr1-61;62Bpa
wt
61
62
βarr1-63;64Bpa
wt
63
64
βarr1-65;66Bpa
wt
65
66
βarr1-67;68Bpa
wt
67
68
βarr1-69;70Bpa
wt
69
70
βarr1-71;72Bpa
wt
71
72
Max Signal [%]
Time [min]
150
100
50
-50
-10
10
20
30
40
50

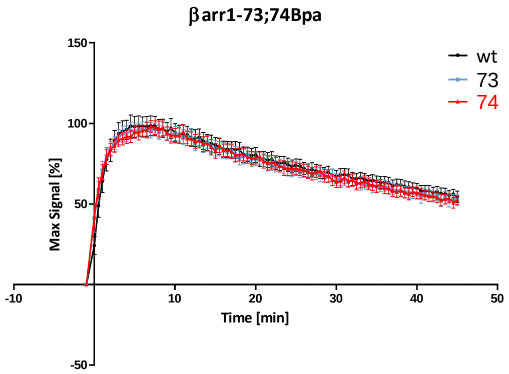
βarr1-73;74Bpa
wt
73
74
Max Signal [%]
Time [min]
150
100
50
-50
-10
10
20
30
40
50

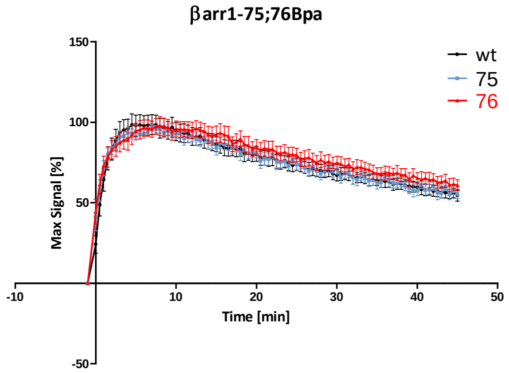
βarr1-75;76Bpa
wt
75
76
Max Signal [%]
Time [min]
150
100
50
-50
-10
10
20
30
40
50

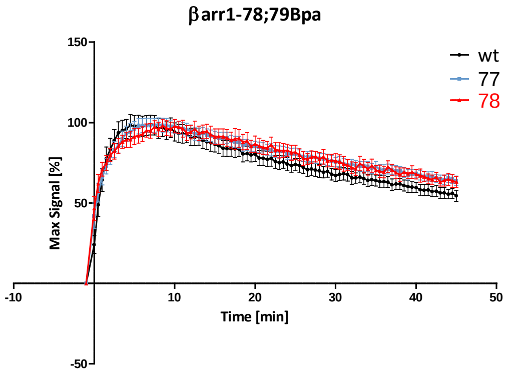
βarr1-78;79Bpa
wt
77
78
Max Signal [%]
Time [min]
150
100
50
-50
-10
10
20
30
40
50

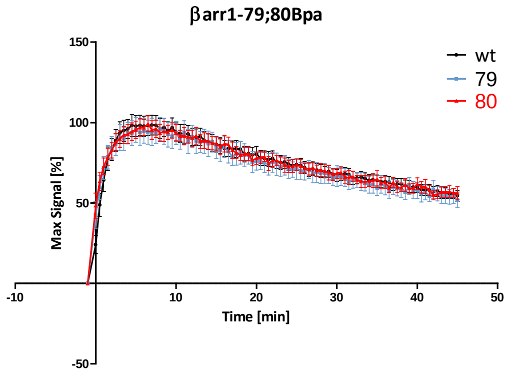
βarr1-79;80Bpa
wt
79
80
Max Signal [%]
Time [min]
150
100
50
-50
-10
10
20
30
40
50

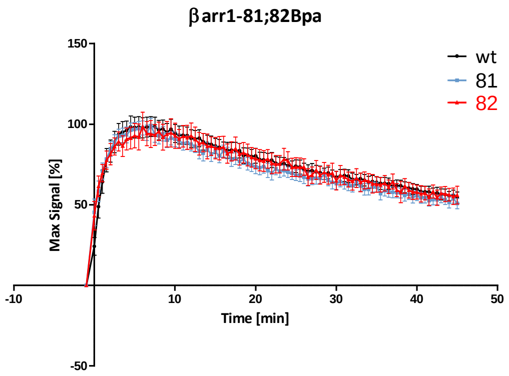
βarr1-81;82Bpa
wt
81
82
Max Signal [%]
Time [min]
150
100
50
-50
-10
10
20
30
40
50

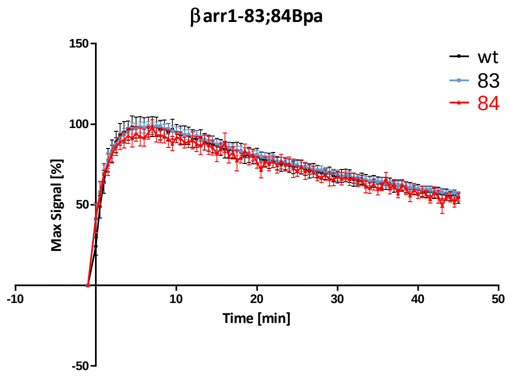
βarr1-83;84Bpa
wt
83
84
Max Signal [%]
Time [min]
150
100
50
-50
-10
10
20
30
40
50

B

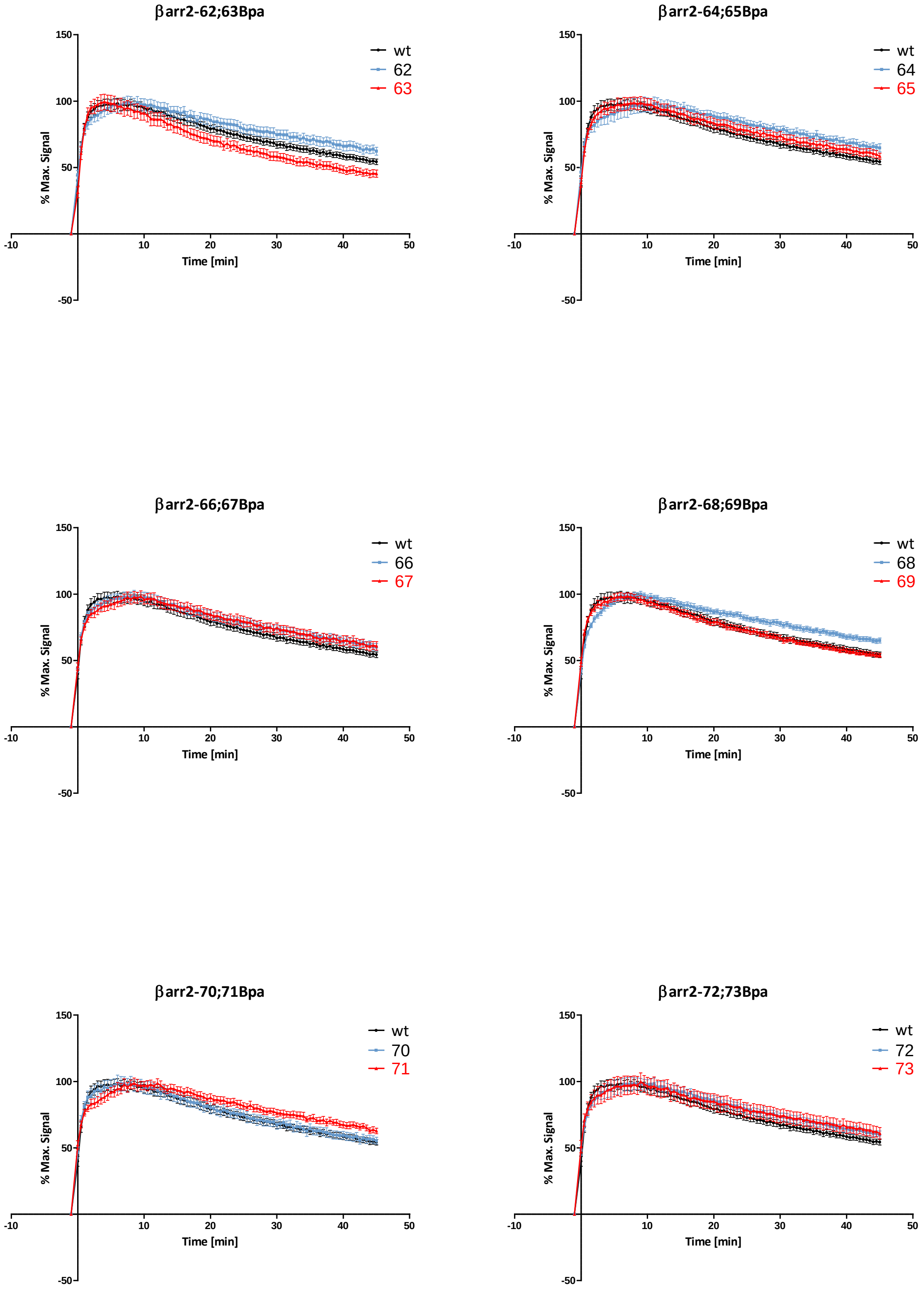
βarr2-62;63Bpa
wt
62
63
βarr2-64;65Bpa
wt
64
65
βarr2-66;67Bpa
wt
66
67
βarr2-68;69Bpa
wt
68
69
βarr2-70;71Bpa
wt
70
71
βarr2-72;73Bpa
wt
72
73
% Max. Signal
Time [min]
150
100
50
-50
-10
10
20
30
40
50

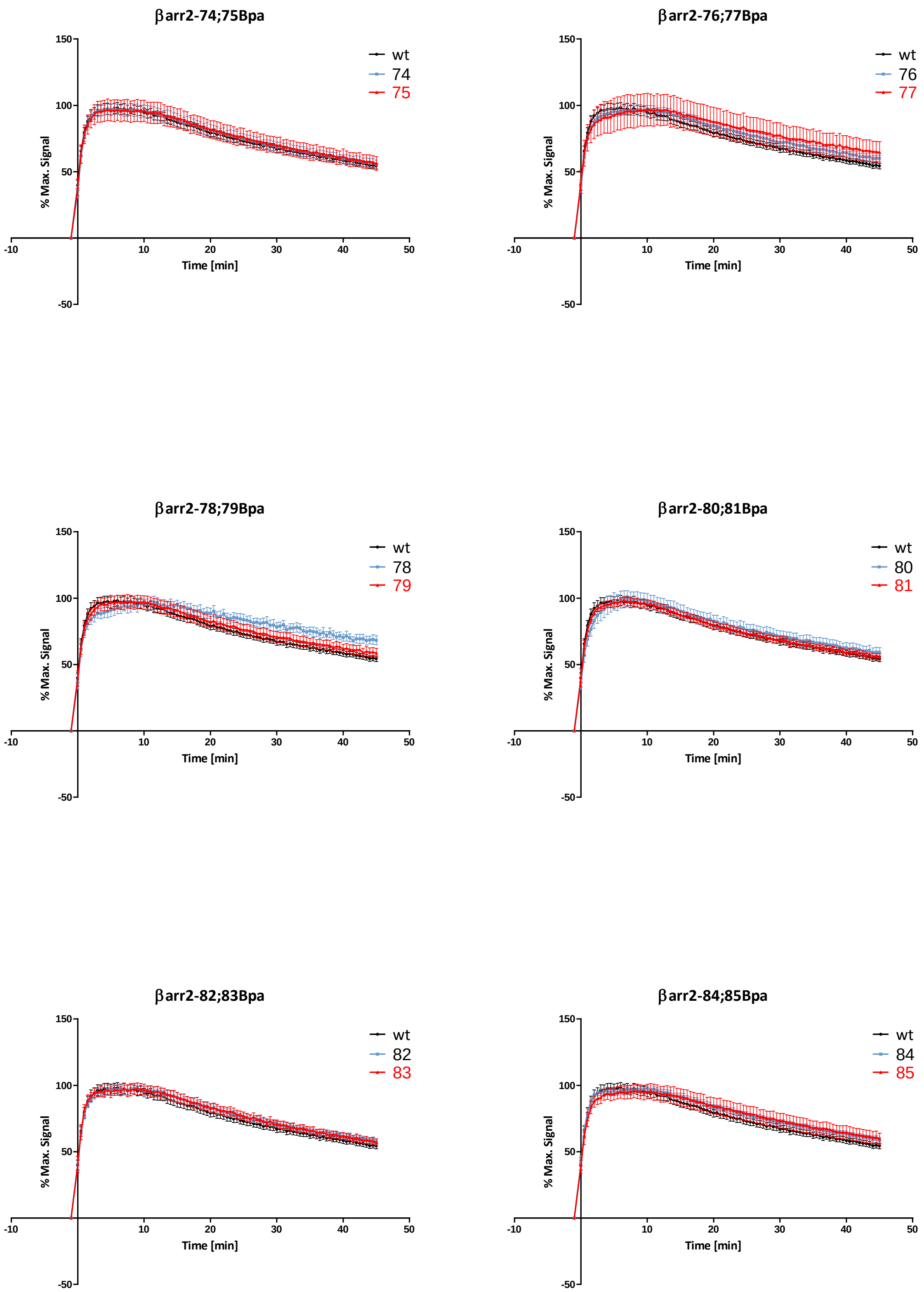

Appendix Figure S1: Recruitment of Bpa-βarrestin mutants to the PTH1R as detected using the NanoBiT® reporter assay (SmBit-Arr/PTH1r-LgBit). 200 nM of pTH(1-34) was added at t=0 and luminescence was measured for 45 min in 0.5 min intervals. All curves were normalized to 100% for the max. signal. Plotted data represent the arithmetic average of three independent experiments, each run in triplicate. Error bars represent the S.E.M. of the biological triplicates.

A NanoBit® reporter assay for βarr1.

B NanoBit® reporter assay for βarr2.

A

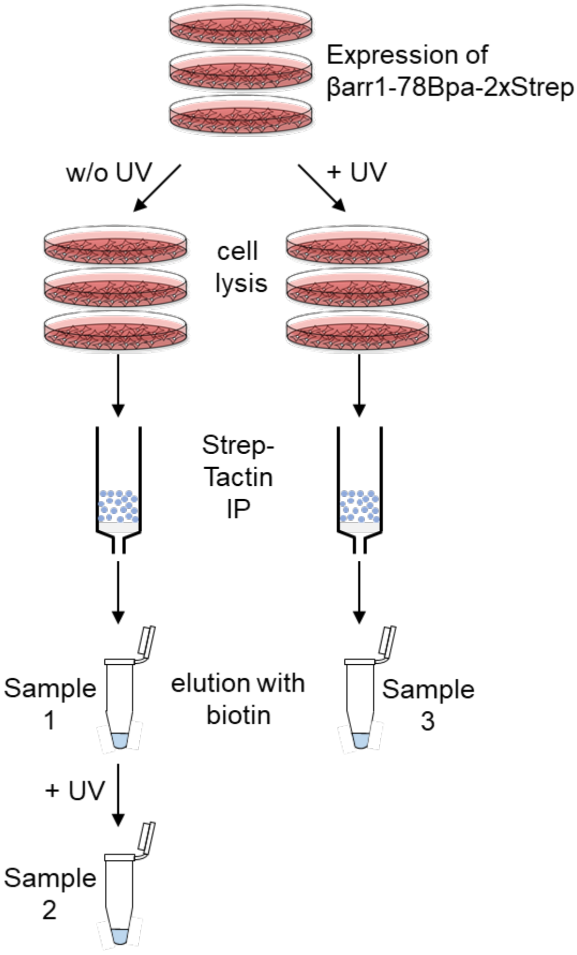

B

MGDKGTRVFK KASPNGKLTV YLGKRDFVDH IDLVDPVDGV VLVDPEYLKE RRVYVTLTCA
FRYGREDLDV LGLTFRKXLF VANVQSFPPA PEDKKPLTRL QERLIKKLGE HAYPFTFEIP
PNLPCSVTLQ PGPEDTGKAC GVDYEVKAFC AENLEEKIHK RNSVRLVIRK VQYAPERPGP
QPTAETTRQF LMSDKPLHLE ASLDKEIYYH GEPISVNVHV TNNTNKTVKK IKISVRQYAD
ICLFNTAQYK CPVAMEEADD TVAPSSTFCK VYTLTPFLAN NREKRGLALD GKLKHEDTNL
ASSTLLREGA NREILGIIVS YKVKVKLVES RGGLLGDLAS SDVAVELPFT LMHPKPKEEP
PHREVPENET PVDTNLIELD TNDDDIVFED FARQRLKGMK DDKEEEEDGT GSPQLNNRKL
WSHPQFEKGG GSGGGSGGSA WSHPQFEK

Appendix Figure S2: Immunoprecipitation (IP) of βarr1-D78Bpa-2xStrep with the Strep-Tactin® XT system and following LC/MS/MS analysis.

A Flowchart of the sample preparation for Figure 3A of the main text.

B Amino acid sequence of βarr1 including the C-terminal double Strep tag. Amino acids identified by PSMs in LC/MS/MS analysis are marked in red. Amino acid 78 of βarr1 (X, marked green) was identified as Bpa.

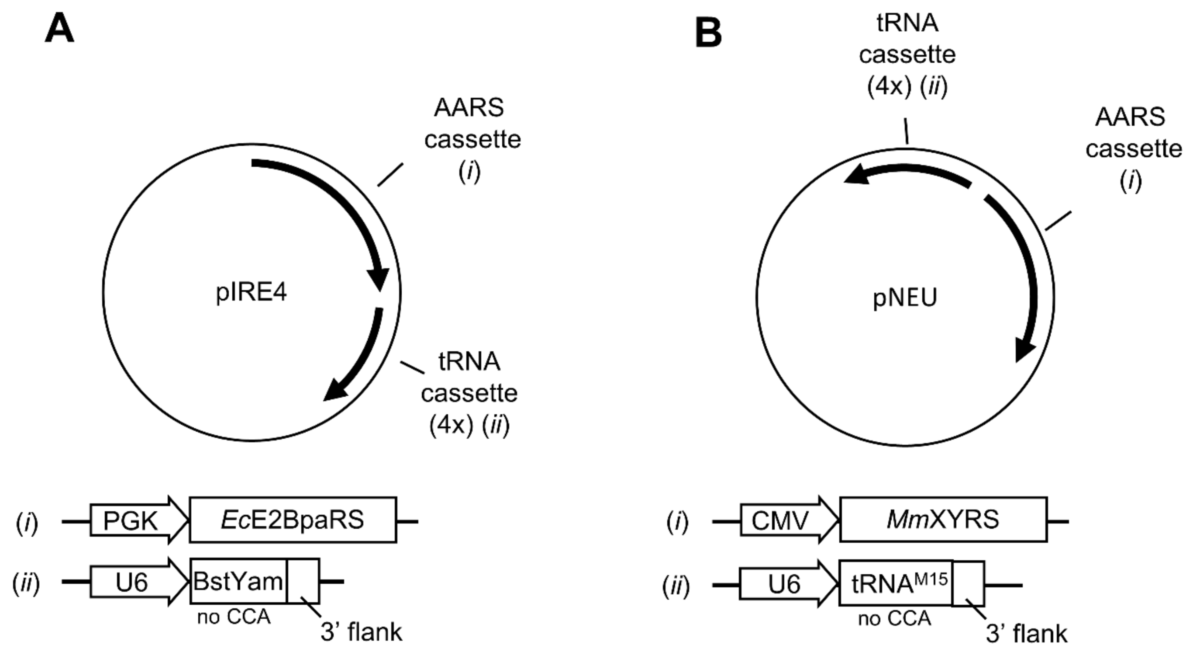

Appendix Figure S3: Plasmids used for the incorporation of ncAAs.

A The backbone of pIRE4 is originally based on pEGFP-N1 (Clontech, Mountain View, CA) and carries a Kan/Neo resistance. The CMV-EGFP sequence of pEGFP-N1 was substituted with the AARS cassette, followed by the tRNA cassettes right downstream the polyadenylation sequence. pIRE4-Bpa contains a humanized gene of the *E. coli* BpaRS under control of a PGK promoter and 4 tandem repeats of a cassette for the expression of the tRNA suppressor from the *Bacillus Stearothermophilus* tRNATyr (BstYam), including a U6 promoter and a 5’-trailer.

B The pNEU backbone is essentially the same as pcDNA3.1 with some variations in the restriction sites. The plasmid contains a humanized gene for the XYPylRS that recognizes BrEtY (evolved from *M. mazei* PylRS) under control of a CMV promoter, as well as four tandem repeats of the enhanced pyrrolysin-tRNA^{M15} gene. The tRNA gene is depleted of the CCA end, is driven by a U6 promoter and followed by a T rich trailer.
