# Supplementary material for: Exploring GPCR‐arrestin interfaces with genetically encoded crosslinkers
Source: EMBO Rep. 2020 Sep 14;21(11):e50437. doi: 10.15252/embr.202050437 (PMC7645262; doi:10.15252/embr.202050437)
Supplement: Supplementary file 2 — Expanded View Figures PDF [file EMBR-21-e50437-s002.pdf]

Expanded View Figures

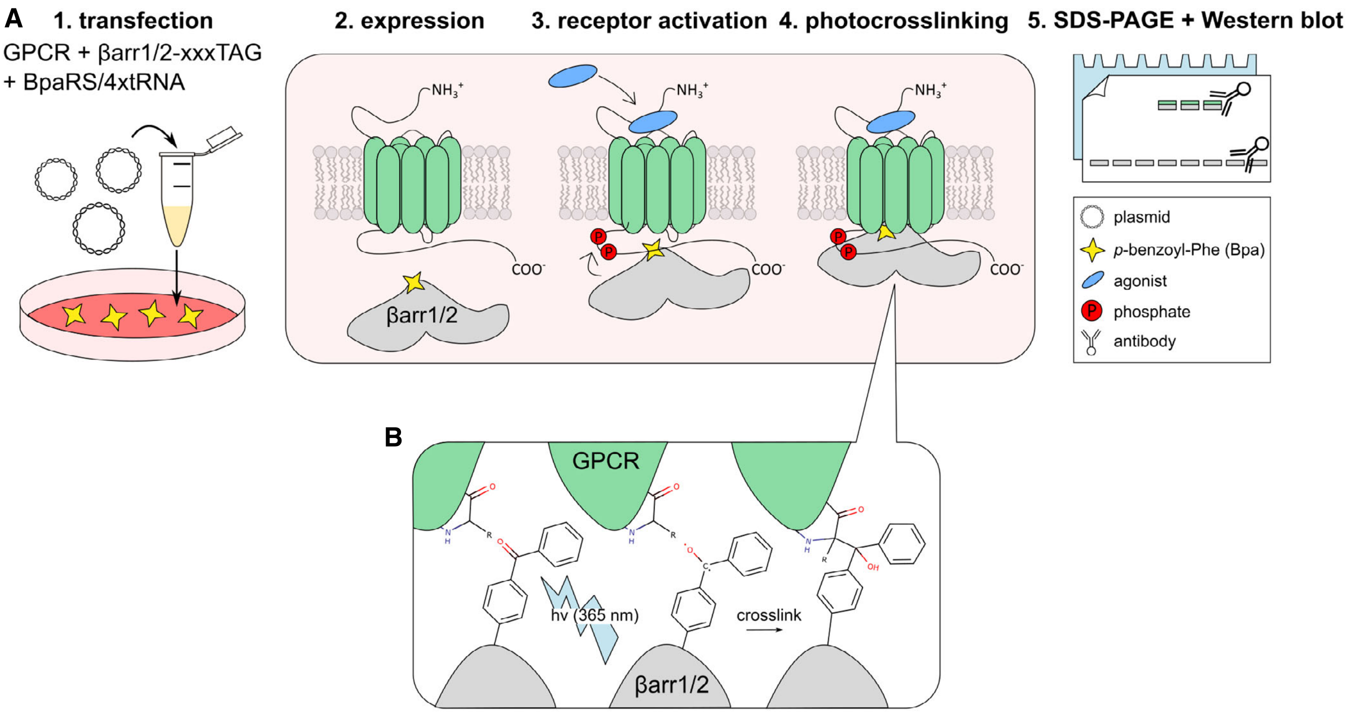

Figure EV1. βarr-GPCR photo-crosslinking.

A 1: Cultured HEK293T cells are supplemented with 250 μM Bpa and transfected with three plasmids. The first plasmid carries the ORF for a GPCR, the second plasmid carries the ORF for one βarr TAG-mutant, and the third plasmid encodes for the tRNA/synthetase pair that incorporates Bpa. 2: The GPCR and the βarr-xxxBpa variant are expressed for 48 h. 3: The GPCR is activated with an agonist for 15 min. 4: The cells are irradiated with UV light (365 nm) for 15 min 5: Samples are lysed, resolved on SDS–PAGE, and the crosslinked arrestin–GPCR complex is detected by immunostaining.

B Mechanism of photo-activation of Bpa. If the diradical species is in close proximity to the GPCR, crosslinking can occur. In general, Bpa inserts into C-H bonds (Dorman & Prestwich, 1994). A covalent arrestin–GPCR complex is formed.

 © 2020 The Authors

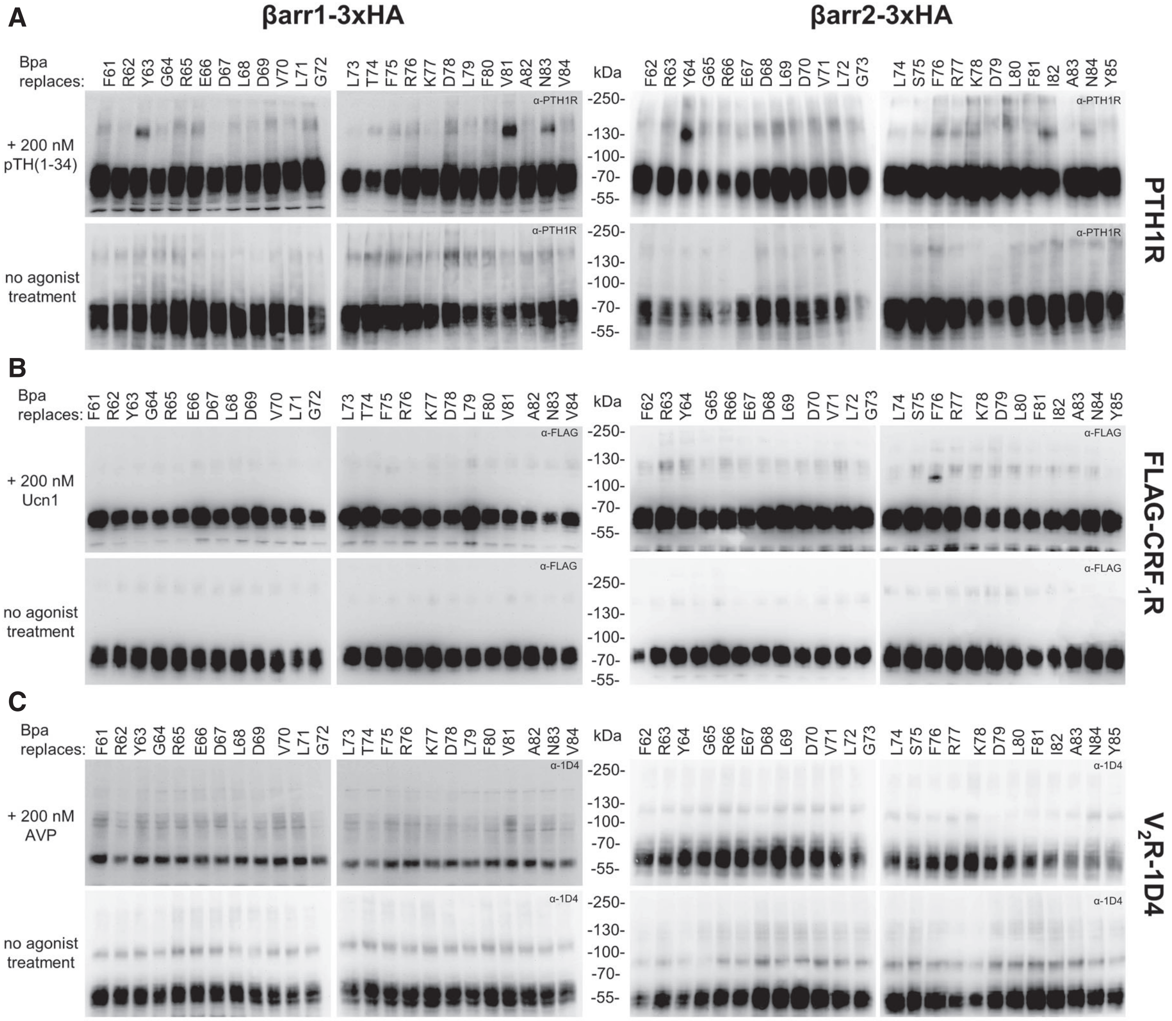

Figure EV2. Photo-crosslinking of Bpa-arrestins with three different GPCRs.

The same samples described in Fig 2 of the main text were immunostained using an antibody detecting the receptor. Overall, crosslinking signals are easier to detect when looking from the side of arrestin with the anti-HA antibody (Fig 2 in the main text).

A PTH1R was detected with a commercial anti-PTH1R antibody, which is very reliable, although it shows some background at about the same size were the crosslinking band is expected.

B CRF_1R was equipped with a FLAG tag at the N-terminus, which was inserted right after the natural signal peptide.

C V_2R was equipped with the 1D4 epitope at its C-terminus.

© 2020 The Authors

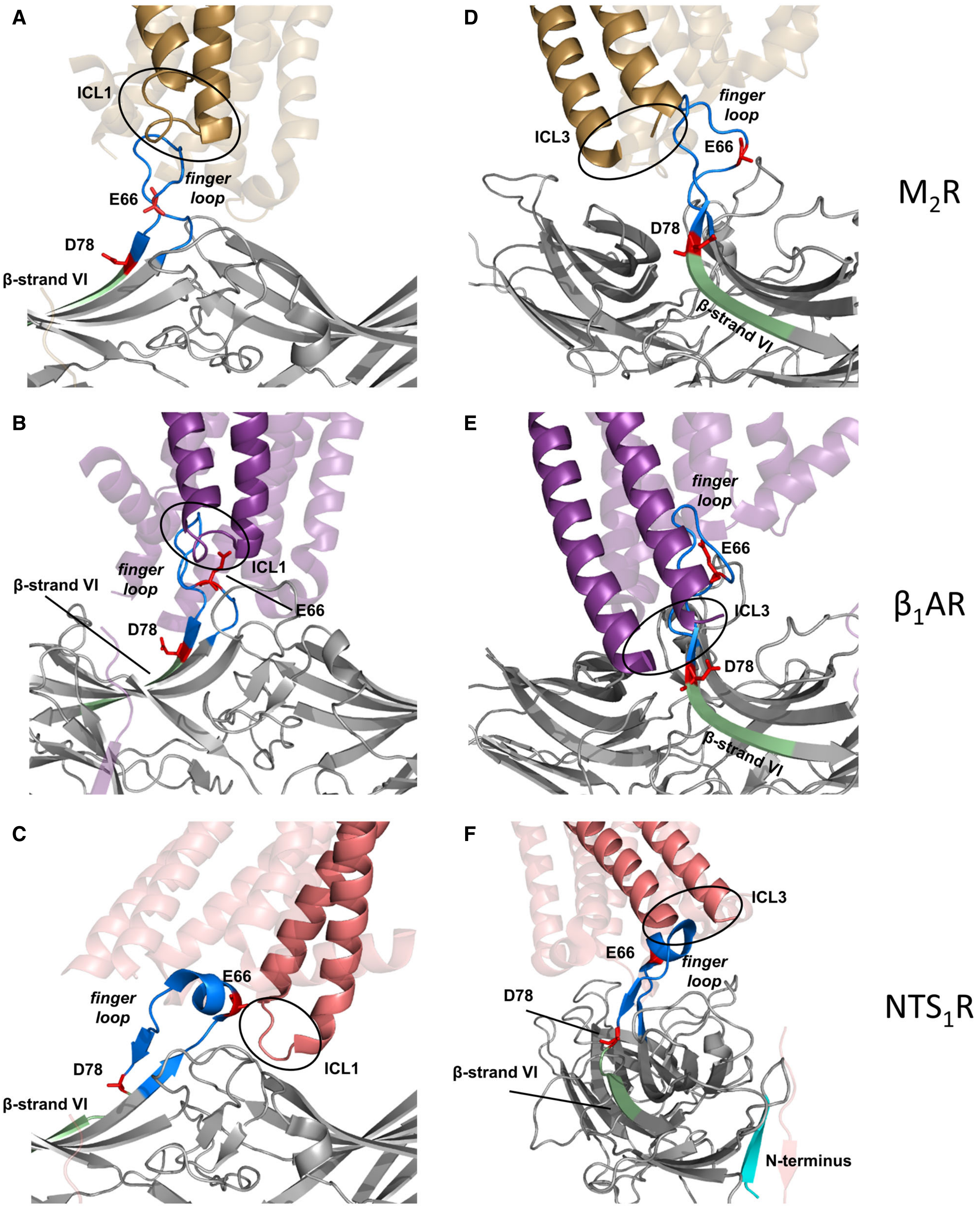

Figure EV3.

 © 2020 The Authors

◀ **Figure EV3. M_2R, β_1AR, and NTS_1R in complex with βarr1.**

Ribbon representation of βarr1 in complex with the M_2R (gold), β_1AR (violet), and NTS_1R (coral) from recent cryo-EM structures (A, D: PDB 6U1N (Staus *et al*, 2020); B, E: PDB 6TKO (Lee *et al*, 2020); C, F: PDB 6PWC (Huang *et al*, 2020)). βarr1 is shown in gray with the *finger loop* in blue and the β-strand VI in pale green as in Fig 1 of the main text. The two main hits of chemical crosslinking with the PTH1R (Fig 5 in the main text), position E66 and D78, are marked in red. βarr1 binds to the M_2R and β_1AR in a similar pose as arr1 to Rho (not shown here), whereas it is rotated by about 90° when docked to the NTS_1R.

A–C These panels focus on the position of the ICL1 in the GPCR–arrestin complex. For the sake of clarity, only the region TM1-ICL1-TM2 in the foreground is fully colored, whereas the rest of the GPCRs is transparent. In all three cases, E66 in the *finger loop* points toward ICL1 of the GPCR, which is compatible with the chemical crosslinking results at the PTH1R.

D–F These panels focus on the position of the ICL3, which is not resolved in any of the structures. For the sake of clarity, only the region TM5-ICL3-TM6 in the foreground is fully colored, whereas the rest of the GPCRs is transparent. The ICL3 of the M_2R and β_1AR is oriented toward β-strand VI, which is compatible with the chemical crosslinking results at the PTH1R, whereas the ICL3 of NTS_1R points toward the arrestin N-terminus (cyan in panel F), away from β-strand VI.

© 2020 The Authors
